# Confinement-Induced One-Dimensional Magnetism in CrSBr Chains via Carbon Nanotube Encapsulation

**DOI:** 10.1021/acsnanoscienceau.5c00162

**Published:** 2026-01-13

**Authors:** Diego López-Alcalá, Alberto M. Ruiz, Andrei Shumilin, José J. Baldoví

**Affiliations:** Instituto de Ciencia Molecular, Universitat de València, Catedrático José Beltrán 2, 46980 Paterna, Spain

**Keywords:** 1D magnetism, CrSBr, carbon nanotubes, spintronics, first-principles

## Abstract

Encapsulating low-dimensional magnetic materials within carbon nanotubes (CNTs) offers a compelling route to stabilize unconventional magnetic states and engineer quantum functionalities at the limit of miniaturization. In this work, we systematically investigate the structural, electronic, and magnetic properties of one-dimensional (1D) CrSBr chains encapsulated within CNTs by using density functional theory (DFT) and spin dynamics simulations. We demonstrate the structural stability of CrSBr@CNT, where confinement and charge transfer cooperate to stabilize ferromagnetism in the 1D limit, which persists up to 50 K. These findings position CrSBr@CNT as a model platform for realizing 1D magnetism and establish CNT encapsulation as a powerful strategy for exploring emergent quantum spin phenomena and engineering nanoscale spintronic devices.

## Graphical abstract

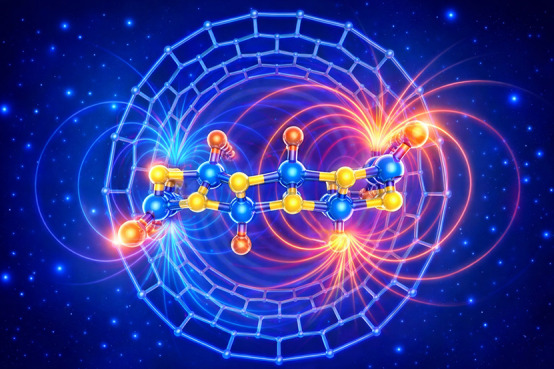

## Introduction

The discovery of intrinsic magnetism in two-dimensional (2D) van der Waals (vdW) materials^1,2^ has opened new frontiers in spintronics^3^ and magnonics,^4^ where low dimensionality and quantum confinement enable emergent spin phenomena and device functionalities.^5^ These 2D magnets show tunable magnetic order with long-range spin transport, and serve as versatile building blocks for assembling vdW heterostructures without chemical constraints.^6,7^ A natural extension of this paradigm is the pursuit of magnetism in the 1D limit, where the confinement of magnetic interactions to a single direction allows for the emergence of exotic phenomena.

1D magnetic systems have already revealed signatures of quantum ballistic transport, anisotropic magnetoresistance, and even the emergence of Majorana fermions.^8–10^ However, their synthesis remains challenging due to strong structural instabilities and curling, which hinder experimental access to their intrinsic properties.^11–14^ In this context, carbon nanotubes (CNTs) emerge as exceptional host structures, providing mechanical stability, chemical inertness and an electrostatic environment conducive to the confinement and protection of 1D chains.^15,16^ Encapsulation via chemical vapor transport (CVT) has proven especially effective,^17^ enabling the realization of robust CNT-based heterostructures where confined 1D phases exhibit unique electronic,^18–20^ catalytic^21,22^ and magnetic properties,^23–27^ which are absent in their bulk or 2D counterparts.^28–35^

Recent experimental efforts have demonstrated that single chains of CrCl_3_ can be confined within CNTs,^27^ exhibiting a spin-glass ground state at 3 K,^23^ while encapsulated 1D β-W and V_x_Te_y_ chains display tunable magnetic configurations.^25,26^ These advances underscore the potential of CNTs to stabilize 1D magnetism. Yet, the number of magnetic systems successfully encapsulated remains limited. Extending this strategy to other magnetic vdW semiconductors with intrinsic anisotropy and scalable synthesis routes remains an open challenge. Among these candidates, CrSBr stands out due to its strong in-plane anisotropy, semiconducting character, and relatively high Curie temperature in the monolayer (*T*_*C*_ = 146 K).^36–38^ Its structural anisotropy, stemming from distinct Cr–S–Cr and Cr–Br–Cr bond geometries, favors elongated growth along the *a* axis and promotes anisotropic spin and charge transport. Importantly, bulk CrSBr crystals can be readily synthesized via CVT,^39^ making them accessible for controlled exfoliation, intercalation, and confinement.^40^

In this work, we investigate the emergence of 1D magnetism in CrSBr nanoribbons (NRs) encapsulated within CNTs using first-principles calculations and atomistic spin simulations. We demonstrate that CNT confinement, combined with charge transfer at the interface, stabilizes robust ferromagnetic order in the 1D limit while preserving the intrinsic anisotropic features of CrSBr. This highlights the applicability of CrSBr in 1D-based materials, where the key properties of the 2D ferromagnetic structure are preserved in the 1D limit. Our results reveal enhanced exchange couplings and coherent magnon propagation, establishing CrSBr@CNT as a prototypical platform for studying confined spin phenomena and for engineering 1D architectures for prospective applications in cutting-edge spin technologies.

## Results

CrSBr is a layered vdW semiconductor with A-type antiferromagnetic (AFM) ordering and pronounced structural, electronic, and magnetic anisotropy. This anisotropy originates from the directional disparity in Cr–X–Cr bonding geometries: along the *a* axis, Cr–S–Cr and Cr–Br–Cr angles are close to 90°, whereas along the *b*-axis, Cr–S–Cr angles approach 160°. In this context, CrSBr monolayers have experimentally shown an unprecedented tendency to grow along the *a* axis, a feature that naturally facilitates the formation of extended 1D architectures. This structural anisotropy has been shown to manifest in axis-dependent carrier transport and spin interactions,^38^ and makes CrSBr a promising platform for dimensional reduction toward 1D magnetism. In parallel, CNTs have emerged as versatile hosts for encapsulating low-dimensional materials, with experimentally accessible inner diameters ranging from 10 to 38 Å.^28^ This size range makes them ideal candidates to accommodate a 1D NR of the magnetic semiconductor CrSBr with a width of 3 to 15 Å (Figure 1a,b).

**Figure 1:**
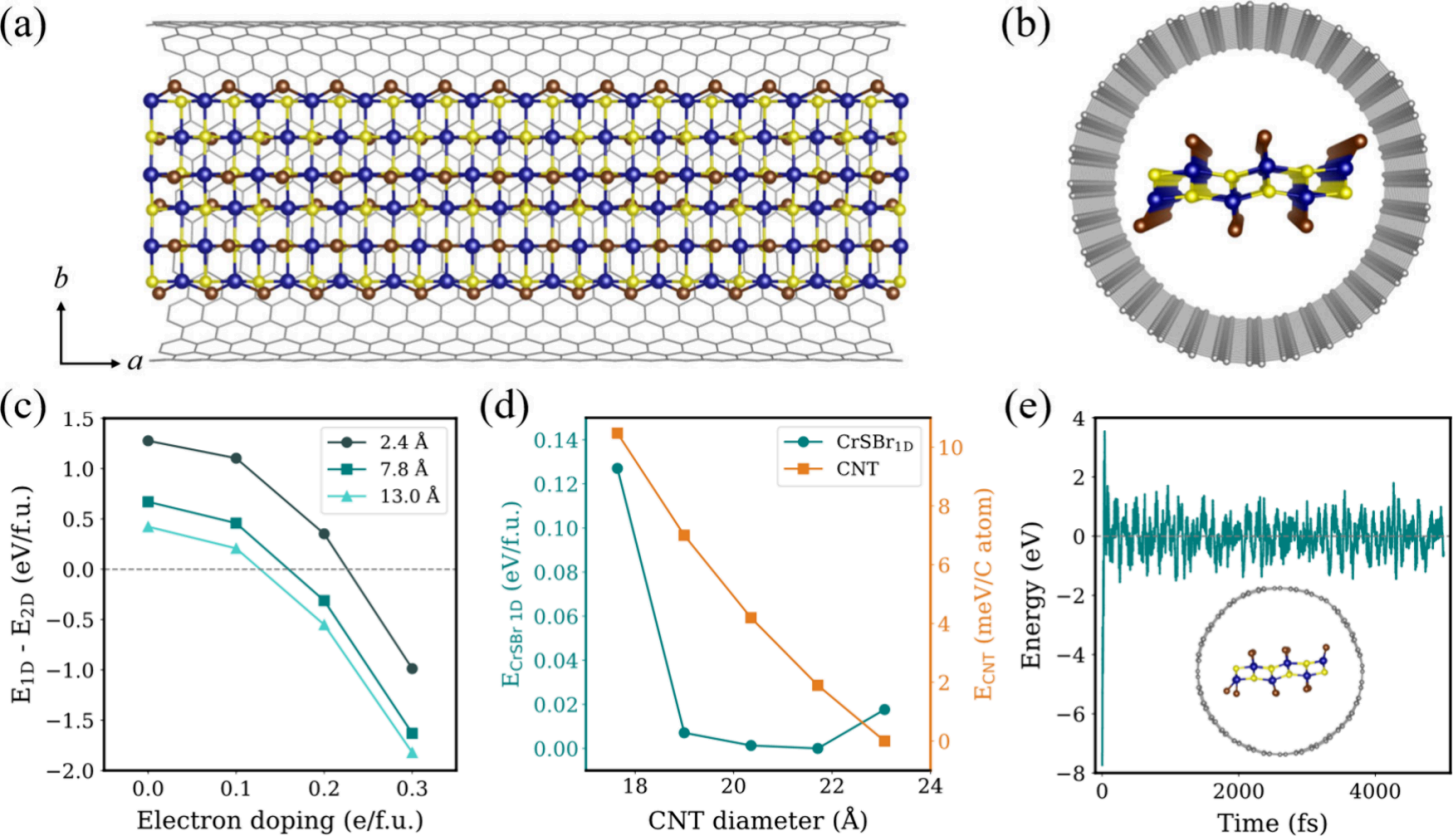
Top (a) and side (b) views of a CrSBr@CNT heterostructure. (c) Stabilization of 1D CrSBr NR with different widths upon electron doping. (d) Relative energy of a separate set of calculations on free-standing CrSBr NR relaxed inside CNTs of varying diameters (left) and the corresponding CNTs (right). (e) AIMD simulation at 300 K in CrSBr@CNT with energies relative to the mean value. Inset image shows a random snapshot of the structure.

To evaluate the stability of 1D CrSBr NRs with respect to their 2D counterparts, we perform density functional theory (DFT) calculations for three different widths of the NRs 2.4, 7.8, and 13.0 Å upon electrostatic doping, revealing that 1D NRs along the *a* direction of CrSBr can be stabilized when the system has an excess of electrons (Figure 1c). This highlights a viable strategy to stabilize a CrSBr NR through a heterostructure, where electrons are transferred from a suitable donor, such as a CNT. Notably, the required electron doping depends sensitively on the ribbon width, since wider ribbons retain characteristics of the 2D parent lattice and require additional doping to reach thermodynamic stability. Based on these results, we focus on a CrSBr 1D NR of 13 Å width for encapsulation into a CNT structure. As hosts, we consider CNTs with diameters ranging from 17.6 to 23 Å, providing sufficient space for hybridization and electron doping for the NR stabilization. We do not consider passivation of dangling bonds in CrSBr NR to simulate experimental conditions in similar 1D@CNT heterostructures,^23,25–27^ where the C skeleton stabilizes the guest chains. With these considerations, we investigate: (i) the use of CNTs with diameters that have been experimentally employed to fabricate similar 1D hybrid nanostructures; (ii) the encapsulation of a sufficiently wide CrSBr 1D chain that can potentially be stabilized; (iii) the effect of CNTs inner cavity on the confinement of 1D nanomaterials.

Consequently, we fully relax the CrSBr@CNT structures described above. Figures 1a and b show the structure of the hybrid system, where CrSBr is only slightly distorted by the confinement, as it preserves its axis-dependent bonding angle. According to our results, the terminal Br atoms tend to approach the carbon skeleton due to the orbital hybridization that emerges at the interface. We first compute separately the total energies of a freestanding CrSBr NR (with the corresponding structure encapsulated in CNTs of different diameters) for comparison (Figure 1d), revealing a noticeable decrease in energy for structures with CNT diameters larger than 18 Å. This behavior arises from the limited space in smaller CNTs, which induces significant distortions in CrSBr and leads to unfavorable conformations (Figure S1 and Table S1). Among these, the structure relaxed inside the armchair (16,16) CNT (21.7 Å diameter) shows the lowest relative energy, likely due to a favorable balance between space filling and hybridization with the carbon skeleton. Therefore, we used this structure as the reference for the following analysis. On the other hand, we observe a general decrease in CNT energy when increasing diameter (Figure 1d). This is because larger CNTs are close to graphene due to lower curvature, but DFT does not consider radial deformations or wall collapse due to mechanical and thermal effects observed experimentally in larger CNTs.^41^ To examine the structural stability of CrSBr@CNT, we performed *ab initio* molecular dynamics (AIMD) simulations (see Methods). Room-temperature simulations (Figure 1e) show that the energy stabilizes, and structural snapshot confirm that the integrity of the system is preserved, supporting the thermodynamic stability of the proposed hybrid CrSBr@CNT structure.

Next, we explore the electronic structure of the hybrid system, as hybridization gives rise to emerging phenomena that significantly alter the electronic and magnetic properties of the heterostructure components. Electronic structure calculations for a 2D monolayer of CrSBr (Figure 2a) reveal a direct band gap at Γ, which is consistent with a semiconducting ground state. Highly dispersive bands are observed along the Γ–Y direction (*b* axis), while they flatten along the Γ–X direction (*a* axis), thus confirming the origin of the pronounced electronic anisotropy in CrSBr. Accordingly, we compute the band structure of the 1D CrSBr NR along the *a* axis (Figure S2) and find that the characteristic electronic dispersion along the Γ–X direction and the semiconducting ground state of the 2D counterpart are preserved. This underscores that the pronounced electronic anisotropy of CrSBr remains robust under dimensional reduction. On the other hand, we simulate the band structures of the different armchair CNTs (Figure 2b and Figure S3), which reveal a metallic ground state characterized by high electron mobility. This is consistent with the extended π-electron system of the carbon skeleton. Figure 2c shows the component-resolved band structure of the CrSBr@CNT heterostructure. One can observe that noticeable charge transfer at the interface shifts the CrSBr (CNT) bands downward (upward). Importantly, beyond the energy shift induced by electron doping from the CNT, we observe only minimal modifications of the CrSBr electronic band structure. This highlights the robust preservation of the intrinsic structural and electronic properties of CrSBr NR after encapsulation. Figure 2c presents the projected density of states (PDOS) of CrSBr@CNT, highlighting that spin polarization occurs exclusively on CrSBr, while the CNT states remain unpolarized. These observations indicate modest interaction between the heterostructure components, which helps maintain their intrinsic electronic properties.

**Figure 2:**
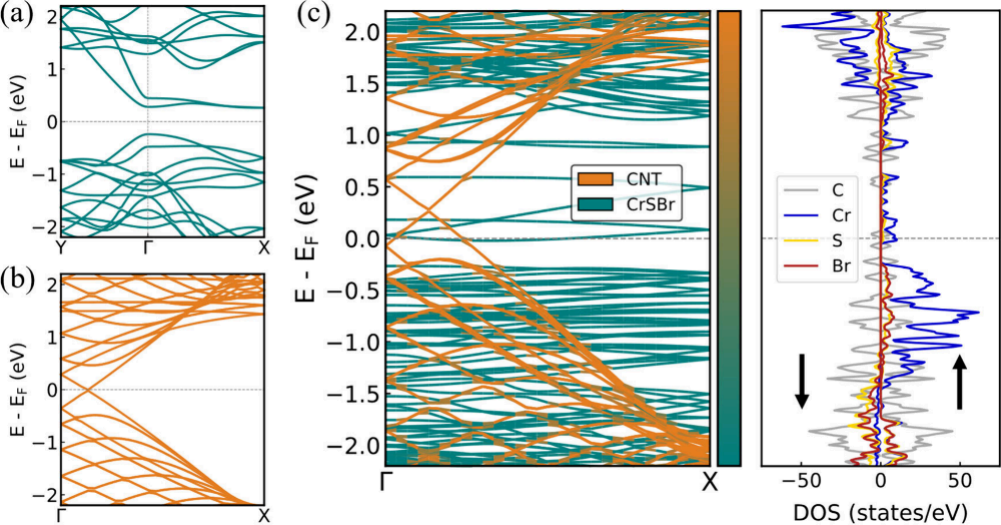
Electronic band structure of (a) 2D CrSBr, (b) (16,16) CNT and (c) CrSBr@CNT with atomic contributions and PDOS.

Orbital hybridization at the heterointerface induces charge transfer from the CNT to the CrSBr NR, as shown in Figure 3a. Bader analysis reveals that the maximum charge transfer occurs for CNTs with diameters of ∼ 20 – 22 Å, reaching up to 0.034 e/CrSBr formula unit (f.u.) (Figure 3b). These results are consistent with previous DFT studies on related systems, involving NRs encapsulated in CNTs, where a discrete charge transfer occurs from the C atoms to the guest material.^24,34^ In contrast, calculations on hybrid heterostructures encapsulating cylindrical chain-like 1D species have reported significantly larger charge transfer ratios,^27,33^ likely due to their geometry, which enhances hybridization within the CNTs through closer interfacial interactions. Figure 3c presents the orbital energy level alignment between the heterostructure components, which refers to an effective energy associated with a given orbital and accounts for its energy distribution and hybridization. These results highlight a noticeable misalignment between CrSBr NR and CNT atoms. This results in discrete charge transfer at the interface (see Section 3 of the Supporting Information). The regions of the CNT closest to the NR atoms are primarily responsible for donating electronic density to CrSBr. This is mainly due to the lower potential barrier along the axial direction of the heterostructure (Figures 3d,e).

**Figure 3:**
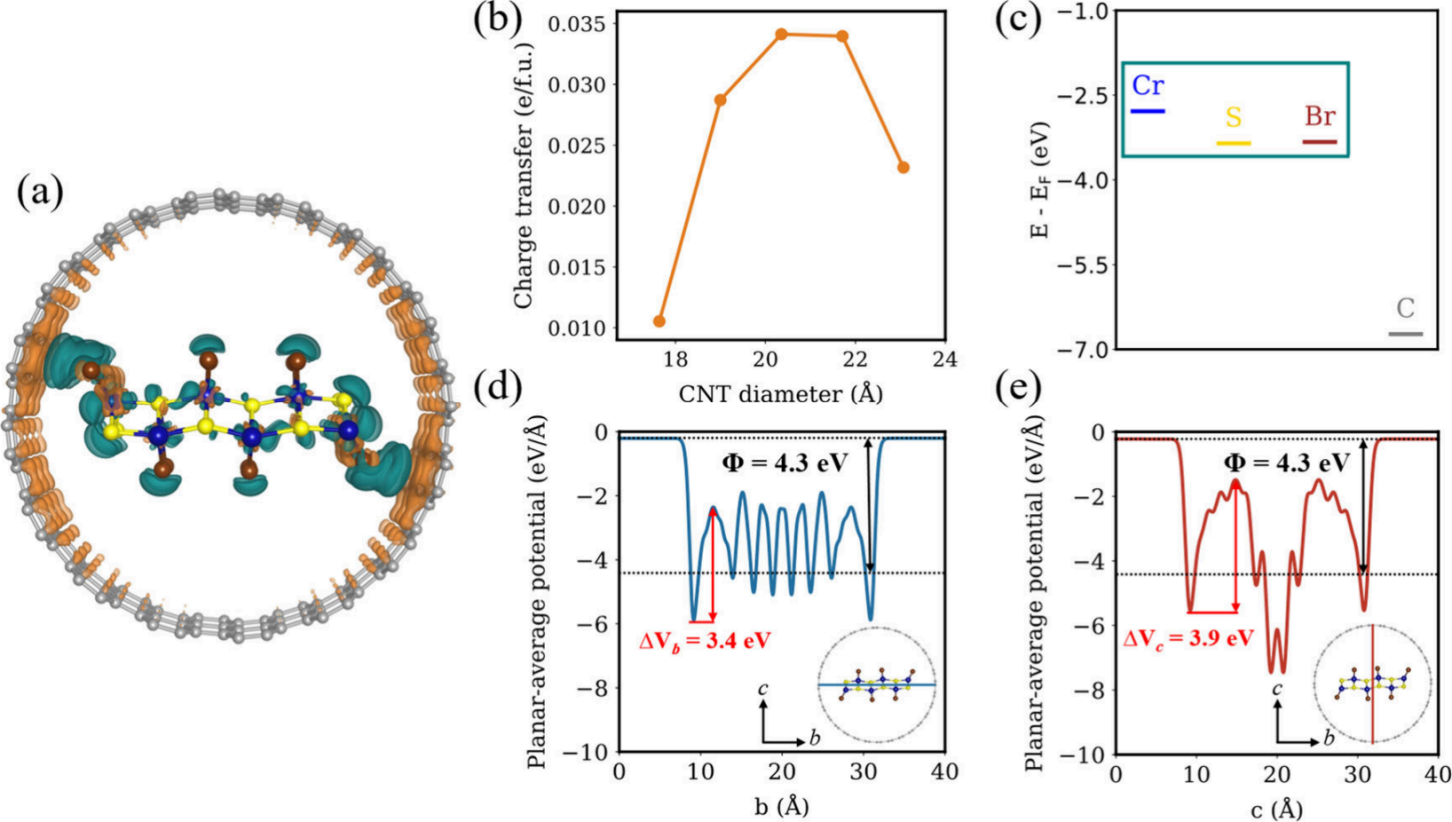
(a) Charge density difference after encapsulation of a CrSBr NR within the CNT. Color code: orange (cyan) represents charge depletion (accumulation). (b) Calculated charge transfer in CrSBr@CNT as a function of CNT diameter. (c) Atom-resolved orbital energy level alignment of the heterostructure. Planar-averaged electrostatic potential along the (d) *b* and (e) *c* directions. Φ denotes the work function.

Then, we examine the magnetic properties of CrSBr@CNT, with particular emphasis on how confinement reshapes the magnetic response of the NR. In the CrSBr monolayer, magnetic Cr atoms couple ferromagnetically, primarily through three nearest-neighbor (NN) exchange interactions: J_1_ (NN along the *a*/*b* directions), J_2_ (NN along *a*), and J_3_ (NN along *b*). Figure 4a presents a schematic illustration of the Js present in a CrSBr NR. The calculated values for each J are reported in Table 1. Interestingly, upon reduction of the dimensionality to the 1D limit, we observe changes in interatomic distances (Table S6) and new magnetic interactions at the NR edges (J_i_’) due to charge redistribution associated with Cr dangling bonds. The inner exchange couplings of the NR closely resemble those of the 2D counterpart as the inner atoms experience less structural distortion and reduced charge transfer from the CNT compared to the outer ones. In contrast, the edge exchange interactions are significantly enhanced, a trend that has also been reported for related 2D magnets like CrI_3_, where edge states play a decisive role in strengthening magnetic couplings.^42,43^ This highlights the importance of unpassivated terminations in the 1D NR, as edge-enhanced magnetic interactions contribute to the stabilization of robust ferromagnetism.

**Figure 4:**
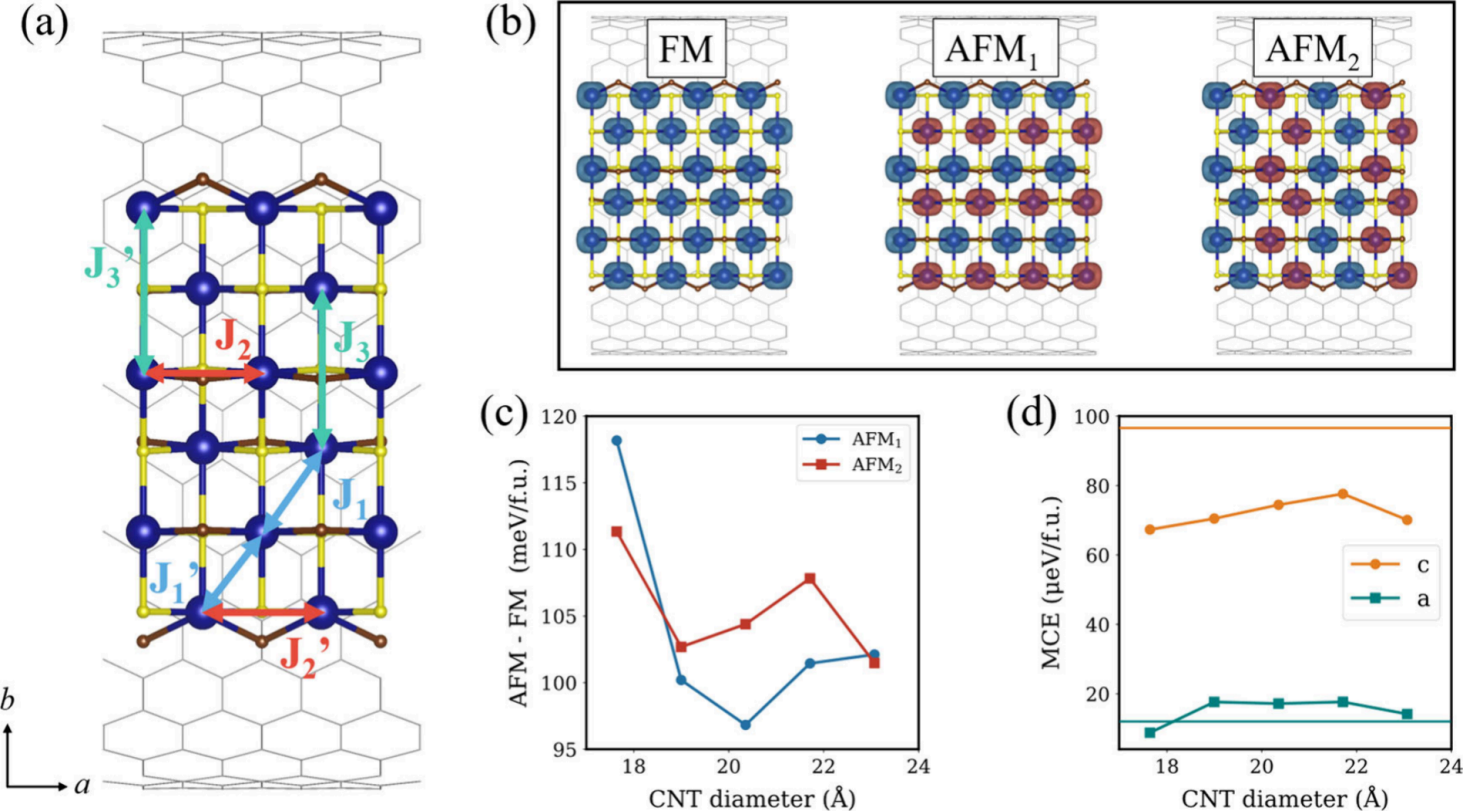
(a) Schematic representation of the exchange interactions (J) in 1D CrSBr encapsulated within CNTs. (b) Spin density distribution for the different magnetic configurations considered. Evolution of (c) AFM – FM energy difference and (d) MCE as a function of CNT diameter. Solid lines in (d) indicate the corresponding MCE values calculated for 2D CrSBr, shown as a reference.

**Table 1:** Magnetic Exchange Couplings (J, in meV) Obtained from DFT+U Calculations for 2D CrSBr, Freestanding 1D CrSBr NR, and Encapsulated CrSBr@CNT

	J_1_	J_1_′	J_2_	J_2_′	J_3_	J_3_′
CrSBr 2D	5.08		3.17		5.58	
CrSBr 1D	4.03	5.27	3.48	7.65	7.25	8.12
CrSBr 1D@CNT	4.00	6.04	3.65	11.27	5.83	10.21

We employ this notation to map a spin Hamiltonian as follows:

1 $$H=-\underset{i\neq j}{\sum }J_{ij}\overset{⃗}{S_{i}\;}\cdot \overset{⃗}{S_{j}\;}-\underset{i}{\sum }K_{i}\overset{⃗}{S_{i}^{2}\;}$$

where *J*_*ij*_ represents the magnetic interaction between magnetic moments (*S*_*i*_ and *S*_*j*_) and *K* is the magnetic anisotropy. In this context, positive (negative) J indicates an FM (AFM) interaction.

After encapsulation within CNTs, we observe a general enhancement of the exchange interactions in CrSBr, mainly driven by charge transfer from the carbon framework to the NR, while the intrinsic structure of the NR remains preserved in the heterostructure. Notably, edge exchange interactions are more strongly affected than the inner ones, in line with the larger magnetic moments found in edge Cr atoms (Table S3). This enhancement arises because edge-localized magnetic states lie closer to the Fermi level (Figure S5), thereby increasing the sensitivity of the edge exchange interactions. To further validate this effect, we simulate the evolution of the Js in pristine 1D CrSBr upon an excess of electrons (Figure S6), finding that the observed trend closely mirrors the behavior obtained for the heterostructure. The strong FM exchange interactions play a pivotal role and are crucial for the robust stabilization of ferromagnetism at the 1D limit in the hybrid CrSBr@CNT system. This robustness is further supported by the large energy separation from competing AFM configurations (Figure 4b), which lie hundreds of meV above the FM ground state (Figure 4c). The magnetic spin state of Cr in the CrSBr@CNT heterostructure could be experimentally elucidated using atomic-resolution STEM–EELS, a technique previously applied to similar CNT-encapsulated 1D chains.^44^ This approach enables the detection and spatial mapping of the spin state of magnetic atoms with atomic-scale resolution.

Regarding magnetic anisotropy in CrSBr, pristine monolayers exhibit an easy axis of magnetization along the *b* crystallographic direction, an intermediate axis along *a*, and a hard axis along *c*.^45^ We calculate magnetocrystalline anisotropy energy (MCE) as MCE = MAE + MSA, where the magnetic anisotropy energy (MAE) originates from spin–orbit coupling (SOC), and the magnetic shape anisotropy (MSA) arises from dipole–dipole interactions. Together, these terms determine the preferred direction of magnetization in the system. Figure 4d shows the calculated MCE for CrSBr encapsulated within different CNTs, clearly demonstrating that the easy and hard magnetization axes remain along the *b* and *c* directions, respectively. Interestingly, the MCE values in CrSBr@CNT closely match those calculated for the pristine 2D monolayer (11 and 95 μeV/f.u. along the *a* and *c* axes, respectively). These results indicate that CrSBr can be feasibly miniaturized to the 1D limit, while preserving its remarkable magnetic properties.

Finally, we conduct spin dynamics simulations using the calculated magnetic parameters of the CrSBr@CNT heterostructure to assess its potential for spintronic applications. Our results reveal a *T*_*C*_ of 50 K (Figure 5a), which is notably lower than that of the CrSBr monolayer. This reduction mainly arises from the dimensionality decrease of CrSBr from 2D to 1D. Although edge exchange interactions are locally enhanced, they cannot compensate for the loss of magnetic neighbors along the truncated direction, leading to a reduced *T*_C_ value compared to the 2D monolayer. Nevertheless, this value is remarkable when compared with reported transition temperatures of 5 K for V_*x*_Te_*y*_ and 3 K for CrCl_3_ 1D chains encapsulated by CNTs.^23,25^ Furthermore, we calculate the magnon dispersion of the CrSBr@CNT hybrid structure using linear spin-wave theory (LSWT)^46^ (Figure 5b). The resulting dispersion consists of six bands originating from size quantization along the *b* direction, corresponding to the acoustic (A) and optical (O) magnon modes. While the first two acoustic branches (A_1_ and A_2_) remain well separated near the Γ point, the third acoustic mode exhibits strong hybridization with the optical magnons (see Figure 5c and Section 6 of the Supporting Information). The acoustic branches closely resemble those observed in the 2D counterpart (Figure S8), thus preserving the distinctive magnetic properties of CrSBr. To examine magnon transport at the macroscopic scale, we simulate the response of extended samples to a localized, short-time magnetic excitation (see Section 7 in the Supporting Information) using the MuMax3 software.^47^ Figures 5d–f show the nonequilibrium magnetization distribution 16 ns after the pulse for CrSBr monolayer, NR, and hybrid CrSBr@CNT heterostructure. Upon dimensional reduction, spin waves become confined along the *a* direction. Excitation generates magnons with a range of wavelengths, where short wavelength magnons exhibit higher group velocities (Figures S9 and S10). Indeed, magnons in CrSBr@CNT propagate faster than in the pristine CrSBr NR, while both 1D systems exhibit higher group velocities than the 2D CrSBr monolayer along the same direction. Therefore, these results support a confinement-driven emergence of 1D magnetism in the CrSBr@CNT.

**Figure 5:**
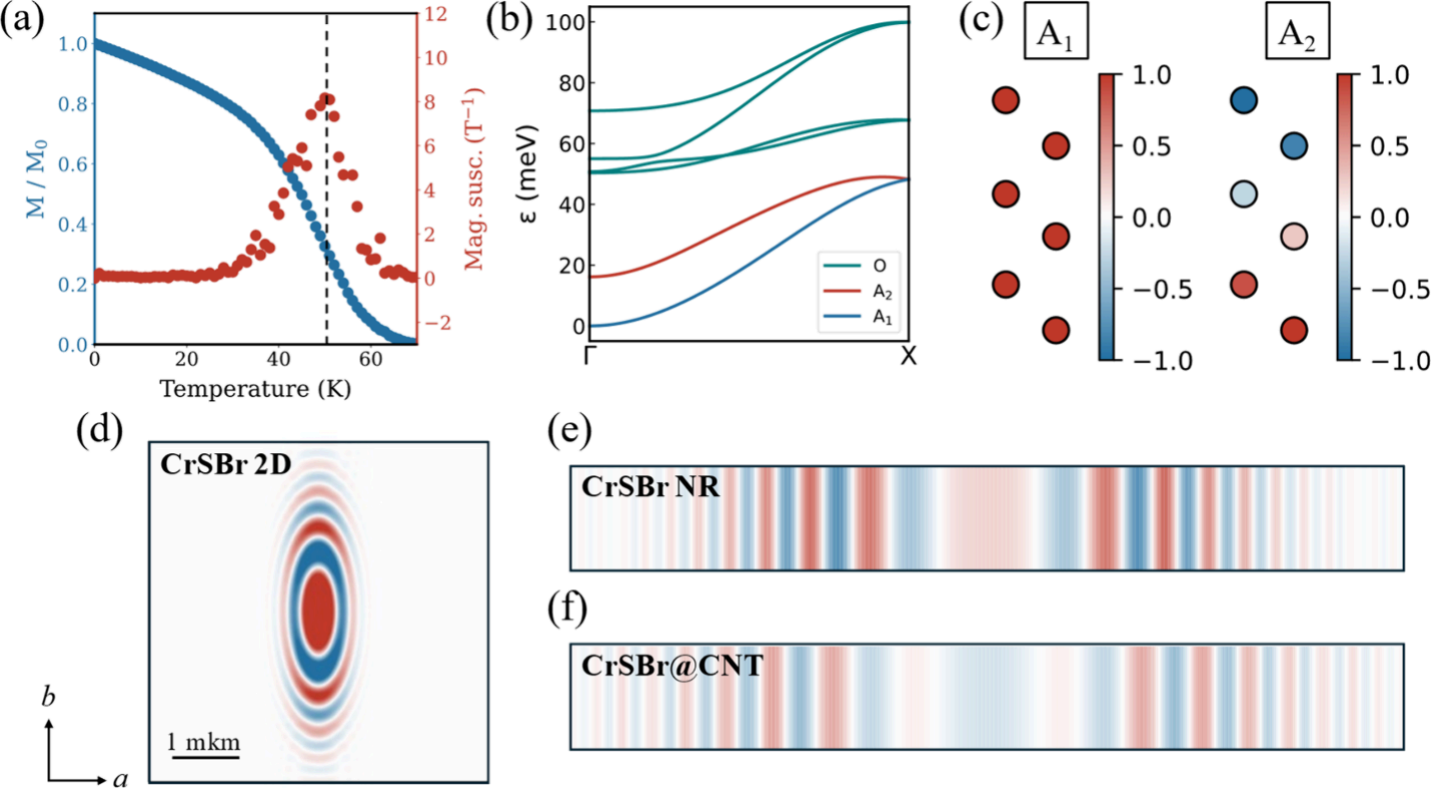
(a) Normalized magnetization (blue) and magnetic susceptibility (red) in CrSBr@CNT as a function of temperature. (b) Simulated magnon dispersion in CrSBr@CNT. Color code: 1st (blue) and 2nd (red) acoustic and optical (cyan) magnon modes. (c) Wavefunctions of the 1st (A_1_) and 2nd (A_2_) acoustic magnon modes respectively on Cr atoms along *b* direction. Macroscopic distribution of magnons excited by a local pulse after 16 ns for (d) 2D CrSBr, (e) 1D CrSBr NR and (f) CrSBr@CNT.

Our results highlight CrSBr as a promising candidate for realizing 1D magnetism via CNT encapsulation. We show that key magnetic properties of CrSBr are preserved under confinement including (i) pronounced electronic anisotropy, (ii) robust ferromagnetic interactions, and (iii) triaxial magnetic anisotropy. Spin dynamics simulations yield a *T*_C_ = 50 K, which is notably higher than values reported for comparable nanostructures.^23,25–27^ Moreover, the methodology developed here can be extended to other 2D magnetic materials synthesized by chemical vapor transport, such as CrPS_4_, providing a versatile route to tailor electronic and magnetic properties in the 1D limit.

In summary, we investigated the feasibility of achieving 1D magnetism in CrSBr by encapsulation within CNTs through first-principles calculations. We demonstrate that the inner cavity of CNTs can effectively host CrSBr NRs, yielding a hybrid heterostructure with enhanced thermodynamic stability driven by charge transfer from the CNT framework. The pronounced structural and electronic anisotropy of CrSBr enables its confinement and facilitates the preservation of its intrinsic magnetic properties down to the 1D limit. This is confirmed by our magnetic exchange and anisotropy calculations, which unveil that robust ferromagnetic correlations persist upon confinement, closely resembling those of their 2D counterpart. Importantly, the interaction with the CNT enhances the magnetic response, leading to a confinement-driven stabilization of ferromagnetism up to 50 K. Furthermore, our spin dynamics simulations predict the propagation of coherent magnons along the confined direction, establishing CrSBr@CNT as a promising platform for exploring 1D magnonics. These findings establish CrSBr as a compelling candidate for 1D magnetism, highlight CNT for engineering quantum-confined magnetic states, and open new avenues for the design of nanoscale spintronic and magnonic devices.

## Methods

DFT calculations were performed using SIESTA code.^48,49^ The generalized gradient approximation (GGA) with Perdew–Burke–Ernzerhof (PBE) parametrization was used to account for exchange-correlation energy^50^ and vdW interactions were considered using the Grimme D3 approximation.^51^ Atomic coordinates of CrSBr NR were relaxed until forces were less than 0.04 eV Å^–1^ and the positions of carbon atoms in the CNTs were kept fixed. A 9 × 1 × 1 *k*-point grid was used to sample the reciprocal space for heterostructure calculations. A vacuum layer of 18 Å was introduced in the *b* and *c* directions to avoid periodic interactions. Double-ζ polarized basis set was used for all atoms in combination with a real-space mesh cutoff of 900 Ry. AIMD simulations were performed using the NVT canonical ensemble for 5 ps with a time step of 1 fs. Norm-conserving fully relativistic pseudopotentials taken from the Pseudo-Dojo database in the psml format were used. Charge transfer at the heterointerface was simulated via Bader charge analysis.^52^ TB2J package is a postprocessing code using a perturbative approach based on nonequilibrium Green’s functions (NEGF) and it was used to calculate magnetic exchange couplings.^53^ For DFT+U calculations, we employed *U*_*eff*_ = 2 eV as discussed in the Section 5 in the Supporting Information. Curie temperature calculations were performed via atomistic spin dynamics simulations as implemented in Vampire software,^54^ where a 600 mm × 30 nm × 30 nm sample was considered in combination with 10 000 equilibration and loop time steps. Micromagnetic real-space simulations were performed using MuMax3 ^47^ (see Section 7 in the Supporting Information).

## Supplementary Material



## Notes

Structural characterization of CrSBr@CNT, electronic structure calculations, orbital energy alignment, magnetic properties, DFT+U, linear spin-wave theory, and micromagnetic simulations (PDF)
