# Supplementary material for: Confinement-Induced One-Dimensional Magnetism in CrSBr Chains via Carbon Nanotube Encapsulation
Source: ACS Nanosci Au. 2026 Jan 13;6(2):208–15. doi: 10.1021/acsnanoscienceau.5c00162 (PMC13087935; doi:10.1021/acsnanoscienceau.5c00162)
Supplement: Supplementary file 1 [file ng5c00162_si_001.pdf]

Supporting Information

Confinement-Induced One-Dimensional Magnetism in CrSBr Chains via Carbon Nanotube Encapsulation

*Diego López-Alcalá[1], Alberto M. Ruiz[1], Andrei Shumilin[1] and José J. Baldoví[1,]**

[1]Instituto de Ciencia Molecular, Universitat de València, Catedrático José Beltrán 2, 46980 Paterna, Spain.

e-mail: j.jaime.baldovi@uv.es

Table of Contents

1. **Structural characterization of CrSBr@CNT** **2**
2. **Electronic structure calculations** **3**
3. **Orbital energy alignment** **4**
4. **Magnetic properties** **4**
5. **DFT+U** **6**
6. **Linear spin-wave theory (LSWT)** **7**
7. **Micromagnetic simulations** **10**

1. Structural characterization of CrSBr@CNT

The first part of this work focuses on designing a suitable structure for the hybrid CrSBr@CNT heterostructure. We selected CNTs with diameters ranging from 17.6 to 23 Å to encapsulate a CrSBr nanoribbon (NR) of 21.7 Å width. Figure S1 shows the relaxed structures, and Table S1 summarizes their key structural parameters. The closest interfacial interaction occurs along the b direction (Δb), where the distance between CrSBr and the CNT decreases to nearly 3 Å in smaller CNTs—for instance, 3.11 Å in a (13,13) CNT—falling outside the typical van der Waals range and thereby destabilizing the heterostructure. The optimized width of a freestanding CrSBr NR is 13.0 Å, closely matching the width of a NR inside (15,15) and (16,16) CNTs, which minimizes distortions and stabilizes the hybrid system. In combination with the results described in the main text, we demonstrate that the (16,16) CNT represents the most suitable structure for encapsulating the CrSBr NR.

Table S1. Closest distances between CrSBr and CNT along the *b* (Δ*b*) and *c* (Δ*c*) directions, and the width of the CrSBr nanoribbon as a function of CNT diameter.

	**(13,13)**	**(14,14)**	**(15,15)**	**(16,16)**	**(17,17)**
**Δb (Å)**	3.11	3.37	3.58	4.01	5.24
**Δc (Å)**	6.4	6.9	7.65	8.26	9.1
**NR width (Å)**	11.88	12.02	12.9	13.62	12.2

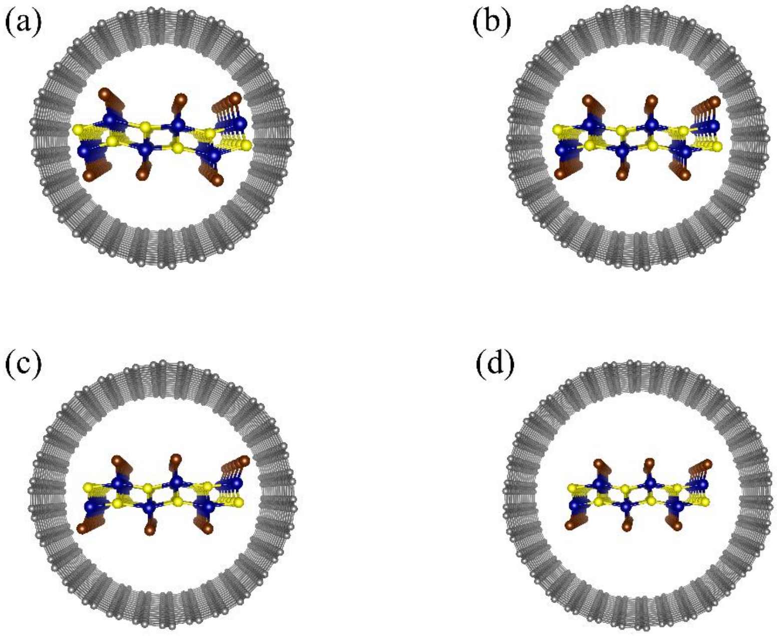

Figure S1. Side view of CrSBr NR encapsulated within (a) (13,13), (b) (14,14), (c) (15,15) and (d) (17,17) CNT.

2. Electronic structure calculations

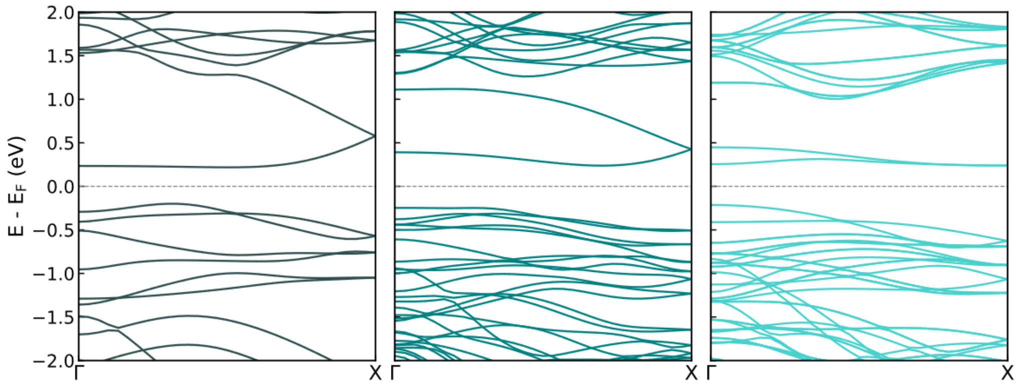

Figure S2. Electronic band structure of CrSBr NR with (a) 2.4, (b) 7.8 and (c) 13.0 Å of width.

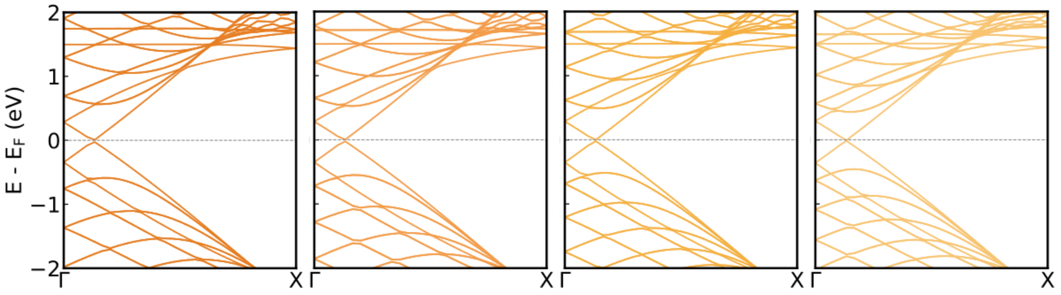

Figure S3. Electronic band structure of (a) (13,13), (b) (14,14), (c) (15,15) and (d) (17,17) CNT.

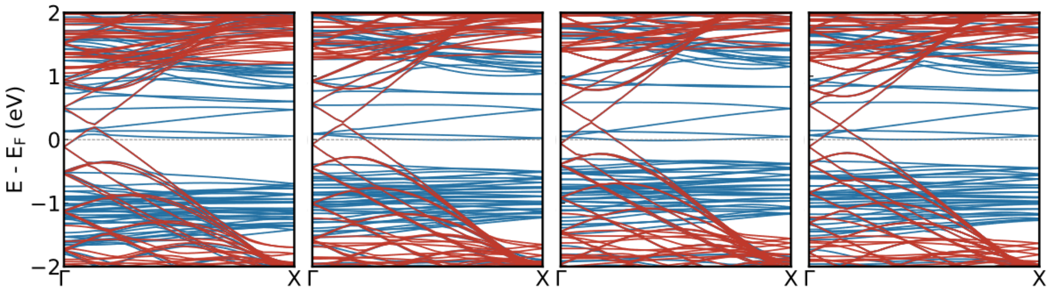

Figure S4. Electronic band structure of a CrSBr NR encapsulated into (a) (13,13), (b) (14,14), (c) (15,15) and (d) (17,17) CNT. Color code: spin up (blue) and down (red).

3. Orbital energy alignment

Orbital energy levels (ε) were calculated to evaluate the energy alignment between them, from which arises the interaction at the heterointerface in CrSBr@CNT. To do that we calculate ε for each component of CrSBr@CNT and for different CNTs diameter as:

$$\varepsilon = \frac{\int_{-\infty}^{E_F} E \cdot g(E) dE}{\int_{-\infty}^{E_F} g(E) dE} \tag{S1}$$

where $g(E)$ indicates the density of states for each atom. Obtained results as a function of CNTs diameter is shown in Table S2.

Table S2. Orbital energy levels (ε) for each component of CrSBr@CNT and for different CNTs diameter. ↑ and ↓ denotes spin up and down, respectively.

CNT	Cr		S		Br		C	
	↑	↓	↑	↓	↑	↓	↑	↓
**(13,13)**	-2.20	-4.08	-3.85	-3.70	-3.38	-3.42	-6.80	-6.80
**(14,14)**	-1.99	-3.91	-3.58	-3.44	-3.20	-3.24	-6.73	-6.73
**(15,15)**	-1.86	-3.80	-3.45	-3.31	-3.10	-3.14	-6.73	-6.73
**(16,16)**	-1.81	-3.76	-3.43	-3.28	-2.90	-3.76	-6.73	-6.73
**(17,17)**	-1.99	-3.92	-3.61	-3.46	-3.18	-3.22	-6.76	-6.76

4. Magnetic properties

We calculate magnetocrystalline anisotropy energy (MCE) as: MCE = MAE + MSA, where MAE represents the magnetic anisotropy energy and MSA represents the magnetic shape anisotropy. MAE is calculated as the energy difference between different spin orientations: $MAE_a = E_a - E_b$ and $MAE_c = E_c - E_b$, where we find the lowest energy when spins are pointing towards *b* axis.

MSA is originated from dipole – dipole interactions and is calculated as following:

$$E_A = \frac{\mu_0}{8\pi} \sum_{i \neq j} \frac{\boldsymbol{m}_i \boldsymbol{m}_j - 3(\boldsymbol{m}_i \boldsymbol{e}_{ij})(\boldsymbol{m}_j \boldsymbol{e}_{ij})}{|\boldsymbol{r}_{ij}|^3} \tag{S2}$$

Here indexes i and j enumerate magnetic atoms, $\boldsymbol{m}_i = g\mu_B \boldsymbol{s}_i$ is magnetic moment of atom i ($\boldsymbol{s}_i$ is spin, in μ_B – Bohr magneton and *g*=2 is g-factor). $\boldsymbol{r}_{ij}$ is the vector distance between the atoms and $\boldsymbol{e}_{ij} = \boldsymbol{r}_{ij}/|r_{ij}|$ is the unit vector in its direction. μ_0 is vacuum magnetic permeability. The long-distance cutoff for the summation was assumed at the distance of 4 unit cells.

Table S3. Calculated magnetic moments in Cr ions in μ_B.

	**Inner Cr**	**Edge Cr**
**CrSBr 2D**	2.96	
**CrSBr 1D**	3.01	3.14
**CrSBr 1D @ CNT**	2.93	3.13

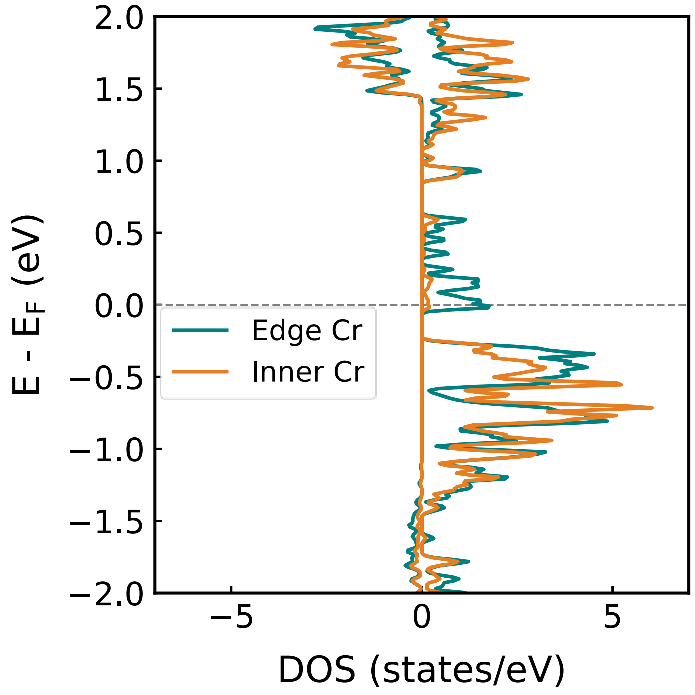

Figure S5. Calculated projected density of states (PDOS) of Cr inner (edge) atoms in CrSBr@CNT represented in orange (cyan).

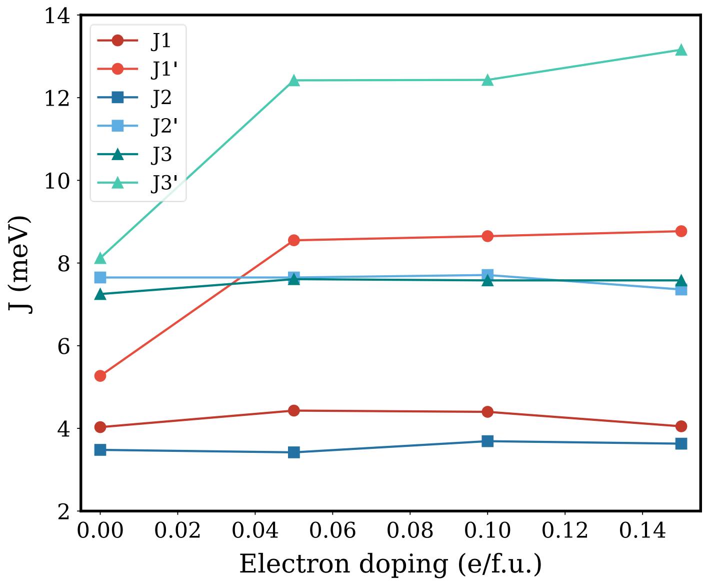

Figure S6. Calculated magnetic exchange couplings (Js) in a free standing CrSBr NR of 13.0 Å width upon electron doping.

Table S4. Magnetic exchange couplings (Js, in meV) calculated in CrSBr@CNT with different CNTs diameter.

CNT	J1	J1'	J2	J2'	J3	J3'
**(13,13)**	5.04	9.51	2.04	8.40	6.80	10.01
**(14,14)**	4.66	5.39	3.05	8.78	6.68	12.44
**(15,15)**	4.17	5.81	3.44	10.58	5.76	10.66
**(16,16)**	4.00	6.04	3.65	11.27	5.83	10.21
**(17,17)**	2.53	5.91	3.04	9.56	6.64	11.87

Table S5. Calculated magnetic anisotropy energy (MAE), magnetic shape anisotropy (MSA) and magnetic crystallographic energy (MCE) along *a* and *c* directions.

CNT	MAE		MSA		MCE	
	a	***c***	***a***	***c***	***a***	***c***
**(13,13)**	37.33	41.17	-29.50	25.39	7.83	66.55
**(14,14)**	45.67	40.58	-28.03	29.92	17.64	70.50
**(15,15)**	45.25	43.58	-28.08	30.88	17.17	74.46
**(16,16)**	45.25	45.75	-27.83	31.64	17.42	77.39
**(17,17)**	41.33	40.58	-27.17	29.52	14.16	70.11

Table S6. Interatomic distance of each J (d_i, in Å).

	d_{J1}	$d_{J1'}$	d_{J2}	$d_{J2'}$	d_{J3}	$d_{J3'}$
**CrSBr 2D**	**3.61**		**3.54**		**4.72**	
**CrSBr 1D**	**3.72**	**3.52**	**3.58**	**3.58**	**4.89**	**4.88**
**CrSBr 1D @ CNT**	**3.62**	**3.47**	**3.51**	**3.51**	**4.69**	**4.86**

5. DFT+U

The Hubbard U parameter critically influences the magnetic properties of transition-metal systems, as it penalizes multiple occupations of d-orbitals and thereby modifies the exchange coupling between magnetic atoms. For CrSBr monolayers, experimental measurements have reported a Curie temperature (T_C) of 146 K and a pronounced triaxial magnetic anisotropy, with the easy axis along the b-direction, the intermediate axis along a, and the hard axis along c. Previous theoretical studies have estimated magnetocrystalline anisotropy energies (MCEs) of 12 and 78 μeV/f.u. along the a and c axes, respectively.

Figures S7 a and b display the calculated MCE values for the a and $c$axes as a function of U_{eff}. A value of $U_{\text{eff}} = 2$ eV yields MCEs of 11 and 95 μeV/f.u. along the a and c axes, respectively, in close agreement with previously reported values. Figure S7c shows the evolution of magnetic exchange interactions Jas a function of U_{eff}, where increasing U suppresses J due to reduced electron hopping between neighboring atoms. The dependence of the calculated T_C on U_{eff} is shown in Figure S7d, revealing excellent agreement with the experimental T_C for $U_{\text{eff}} = 1 \sim 2$ eV. Based on these results, a value of $U_{\text{eff}} = 2$ eV was selected for the present study, as it provides a reliable description of the experimentally observed magnetic behavior of CrSBr.

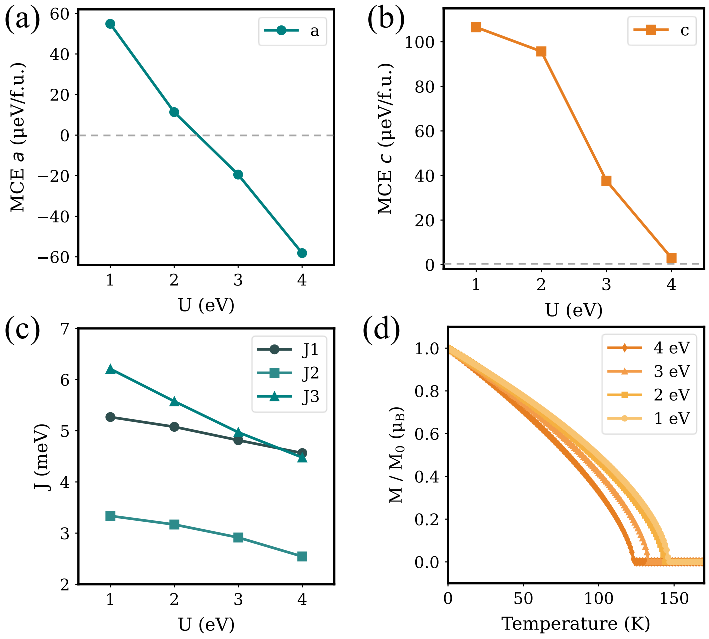

Figure S7. Magnetic crystallographic energy (MCE) in (a) *a*, (b) *c*, (d) magnetic exchange interactions (J) and (d) Curie temperature with different U values calculated in CrSBr monolayer.

6. Linear spin-wave theory (LSWT)

Figure S8(a) presents the magnon dispersion of 2D CrSBr along the high-symmetry Γ–X–S–Y–Γ path. The dispersion exhibits two branches — acoustic (A) and optical (O) — which become degenerate along the X–S–Y segment of the k-path. Figures S8(b) and S8(c) show the x- and y-components of the magnon group velocity along the Γ–X and Γ–Y directions, respectively. The group velocity of the acoustic magnons remains positive throughout, reaching up to 1.2×10^4 m/s in the x-direction and 1.8×10^4 m/s in the y-direction. In contrast, optical magnons exhibit negative group velocity over substantial portions of the k-path, corresponding to regions where their energy decreases with increasing wavevector.

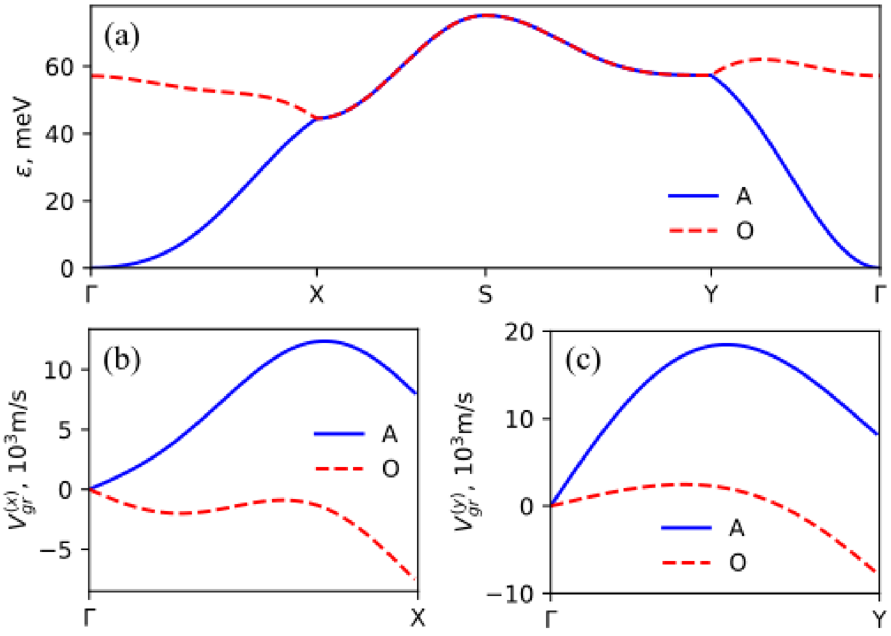

Figure S8. Magnon properties of 2D CrSBr. (a) magnon spectrum along the high-symmetry k-path. (b) Group velocity of the magnons propagating in x-direction. (c) Group velocity of the magnons propagating in y-direction.

Figure S9 illustrates the magnon characteristics of a 1D CrSBr nanoribbon. The quantization of the wavevector along the y-direction results in three acoustic (A1–A3) and three optical (O1–O3) magnon branches at small wavevectors. Figure S9(c) presents the corresponding wavefunctions of all branches at the Γ-point. At larger k-values, hybridization between acoustic and optical modes occurs, giving rise to anticrossing behavior. Consequently, the A2 branch exhibits negative group velocity at high wavevectors, as shown in Figure S9(b).

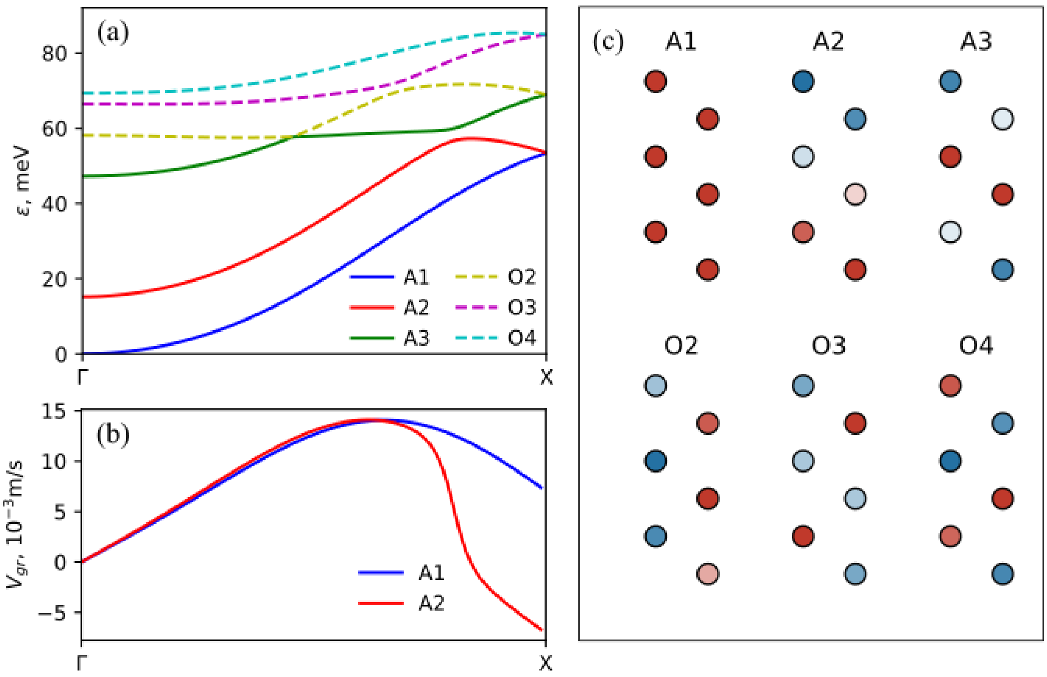

Figure S9. Magnon properties of 1D nanoribon of CrSBr. (a) magnon dispersion (b) Group velocity acoustic branches A1 and A2. (c) Magnon wavefunctions at the Γ-point.

Figure S10 presents the magnon characteristics of the CrSBr@CNT heterostructure. The overall dispersion is qualitatively similar to that of the 1D CrSBr nanoribbon. However, the third magnon branch exhibits hybridization with optical modes already at the Γ-point; thus, the spectrum can be described as comprising two acoustic and four optical branches. Notably, the maximum group velocity of the A1 branch (1.2×10^4 m/s) is slightly lower than that of the pristine 1D nanoribbon (1.4×10^4 m/s). Nevertheless, encapsulation within the carbon nanotube enhances the group velocities of low-k magnons, as illustrated in Figure S10(b).

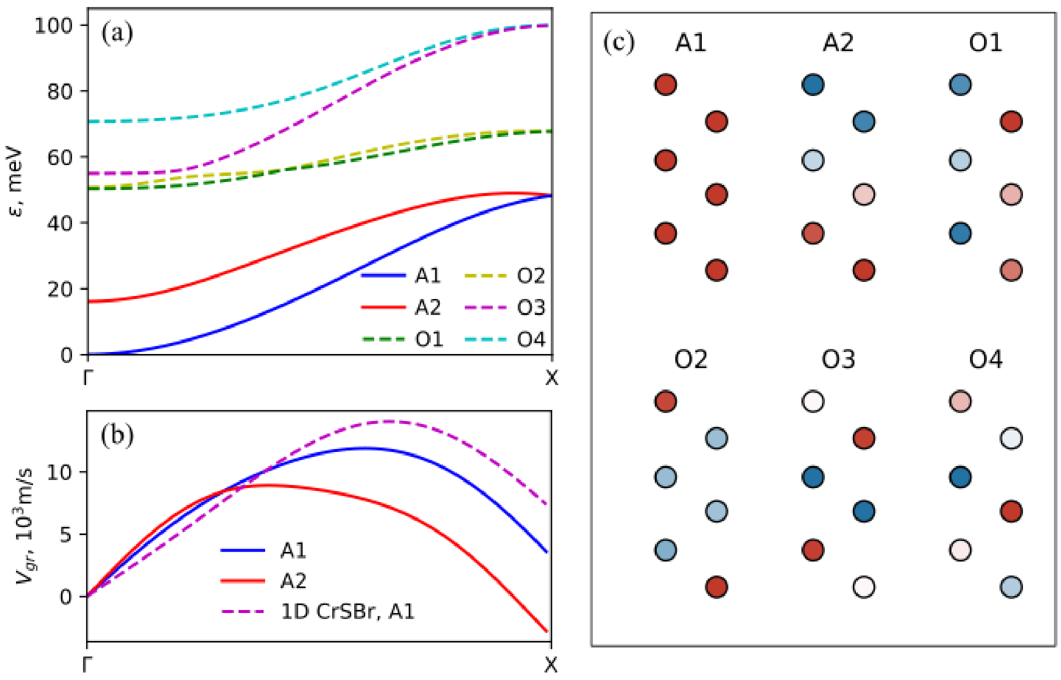

Figure S10. Magnon properties of CrSBr@CNT. (a) magnon dispersion (b) Group velocity acoustical branches A1 and A2 compared with group velocity of A1 magnon branch of 1D CrSBr nanoribbon. (c) Magnon wavefunctions at the Γ-point.

7. Micromagnetic simulations

The micromagnetic simulations were performed in MuMax3 package with the exchange stiffness tensor derived from spin Hamiltonian with the expression:

$$A_{\alpha\beta} = \frac{1}{V_{uc}} \sum_{i,j} J_{ij} S_i S_j \frac{r_{ij}^{(\alpha)} r_{ij}^{(\beta)}}{2} \tag{S3}$$

Here α and β enumerate the Cartesian components of the tensor, i and j enumerate magnetic atoms. The summation over i is performed within a unit cell while j enumerates all the atoms connected to the atom i with exchange interaction J_{ij}. $r_{ij}^{(\alpha)}$ is an α Cartesian component of a radius-vector between atoms i and j. V_{uc} is the unit cell volume. The anisotropic exchange stiffness $A_{\alpha\beta}$ is introduced into MuMax3 as a custom exchange field.

Micromagnetic simulations were performed for 1D CrSBr and CrSBr@CNT samples of 5 μm length, and for a 2D CrSBr square with lateral dimensions of 5 μm × 5 μm. The initial magnetization was oriented along the y-axis, with a stabilizing magnetic field of 0.1 T applied in the same direction. The excitation was introduced as a local field-like torque along the x-direction with a maximum amplitude of 0.01 T. The driving pulse had an angular frequency of 10.65 rad ns^{-1} and a Gaussian envelope with a half-width of 0.59 ns. The simulation commenced approximately 2 ns before the maximum of the pulse. The simulated values of m_x at different time intervals after the beginning of simulation are shown in Figure S11.

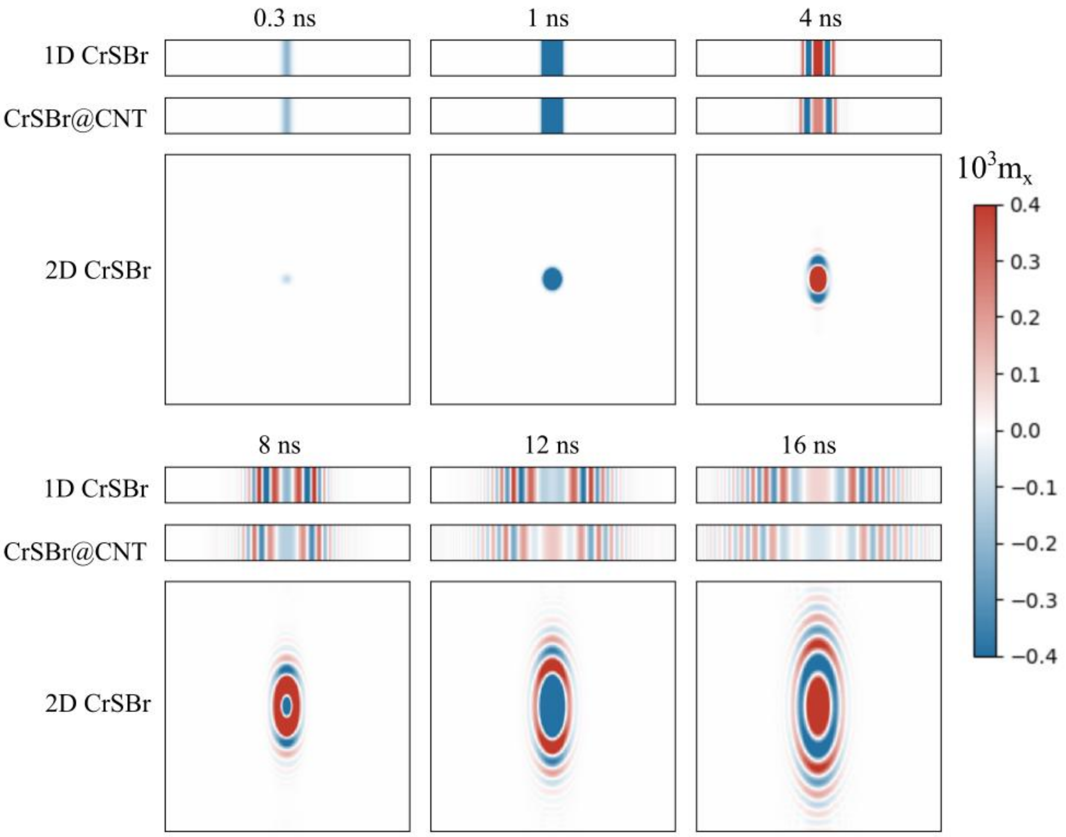

Figure S11. Distributions of the magnetization m_x at different delays after the start of the simulation.
